# Supplementary material for: Histidine: A Systematic Review on Metabolism and Physiological Effects in Human and Different Animal Species
Source: Nutrients. 2020 May 14;12(5):1414. doi: 10.3390/nu12051414 (PMC7284872; doi:10.3390/nu12051414)
Supplement: Supplementary file 1 [file nutrients-12-01414-s001.zip › Methodology_supplementary material.docx]

**Methodology for identifying source documents for systematic review on histidine: metabolism and physiological effects in human and different animal species**

1. General search strategy:

Step 1: Search PubMed with key words in title or abstract.

Step 2: Refine results of Step 1 by individually screening each result to identify articles relevant to topic.

Step 3: Check “similar articles” list in PubMed for entries identified in Step 2.

Step 4: Acquire copies of relevant articles from Steps 2 and 3.

Step 5: Identify additional relevant articles from references cited in articles acquired in Step 3.

Step 6: Acquire copies of relevant articles from Step 5.

Step 7: Add citations for relevant articles from Steps 2-6 to database in citation manager (Zotero).

Step 8: Review individual articles and decide which ones have sufficient relevant information to cite in article.

Section I: Metabolism:

Pubmed search:

("histidine"[MeSH Terms] OR "histidine"[All Fields]) AND HCL[All Fields] AND ("metabolism"[Subheading] OR "metabolism"[All Fields] OR "metabolism"[MeSH Terms] OR "metabolism"[All Fields] OR "metabolic networks and pathways"[MeSH Terms] OR ("metabolic"[All Fields] AND "networks"[All Fields] AND "pathways"[All Fields]) OR "metabolic networks and pathways"[All Fields])

Initial results: 126; 19 articles judged relevant to topic.

Section II: Human:

Pubmed search:

(histidine[Title/Abstract] OR histidine-HCL [Title/Abstract]) AND (nutrition[Title/Abstract] OR

diet[Title/Abstract] OR dietary[Title/Abstract]) NOT (amino acid[Title/Abstract] OR chicken[Title/Abstract] OR chick[Title/Abstract] OR pig[Title/Abstract] OR mice[Title/Abstract] OR

fish[Title/Abstract] OR rat[Title/Abstract] OR ruminant[Title/Abstract])

("humans"[MeSH Terms] AND English[lang])

Initial results: 552; 21 articles judged relevant to topic.

Section III: Rats:

Pubmed search:

("histidine"[MeSH Terms] OR "histidine"[All Fields]) AND ("diet"[MeSH Terms] OR "diet"[All Fields]) AND ("rats"[MeSH Terms] OR "rats"[All Fields])

Initial results: 395; 14 articles judged relevant to topic.

Section IV: Pigs:

Pubmed search:

("histidine"[MeSH Terms] OR "histidine"[All Fields]) AND ("diet"[MeSH Terms] OR "diet"[All Fields]) AND ("swine"[MeSH Terms] OR "swine"[All Fields] OR "pigs"[All Fields])

Initial results: 101; 10 articles judged relevant to topic.

Section IV: Chicken:

Pubmed search:

(histidine[Title/Abstract] OR histidine-HCL [Title/Abstract]) AND (nutrition[Title/Abstract] OR diet[Title/Abstract] OR dietary[Title/Abstract]) AND chicken [All fields]

Initial results: 81; 10 articles judged relevant to topic.

Section V: Fish:

("histidine"[MeSH Terms] OR "histidine"[All Fields]) AND ("diet"[MeSH Terms] OR "diet"[All Fields]) AND ("fish"[MeSH Terms] OR "fish"[All Fields])

Initial results: 60; 10 articles judged relevant to topic.

Section VI: Ruminants:

Pubmed search:

("histidine"[MeSH Terms] OR "histidine"[All Fields]) AND ("diet"[MeSH Terms] OR "diet"[All Fields]) AND ("ruminants"[MeSH Terms] OR "ruminants"[All Fields])

Initial results: 90; 10 articles judged relevant to topic.

**9 articles were added on the basis of reviewers’ remarks that did not appear in the bibliographical search.**

Section I: Metabolism: 7 articles were added

Section III: Rats: 1 article was added

Section V: Fish: 1 article was added

2. Data extraction:

The results of data on metabolism and physiological effects of histidine in different animal species on the following outcomes were extracted: plasma histidine, food intake, body weight, taste acuity, smell acuity, memory disorders, metal ion status, skin dysfunction, insulin resistance, inflammation, neuroprotection, growth, cholesterol, cancer, toxicity, gain-to-feed, anti-oxidant role, buffering role, cataract, protein synthesis. Additional data on study design, species, histidine form and supply were extracted and taken into account for data analysis.
