# Supplementary material for: Histidine: A Systematic Review on Metabolism and Physiological Effects in Human and Different Animal Species
Source: Nutrients. 2020 May 14;12(5):1414. doi: 10.3390/nu12051414 (PMC7284872; doi:10.3390/nu12051414)
Supplement: Supplementary file 1 [file nutrients-12-01414-s001.zip › manuscript.v13.pdf]

nutrients

Review

Histidine: A Systematic Review on Metabolism and Physiological Effects in Human and Different Animal Species

Joanna Moro [1], Daniel Tomé [1], Philippe Schmidely [2], Tristan-Chalvon Demersay [3] and Dalila Azzout-Marniche [1,*]

[1] Université Paris-Saclay, AgroParisTech, INRAE, UMR PNCA, 75005 Paris, France; joanna.moro@agroparistech.fr (J.M.); tome@agroparistech.fr (D.T.)
[2] Université Paris-Saclay, AgroParisTech, INRAE, UMR0791 Mosar, 75005 Paris, France; philippe.schmidely@agroparistech.fr
[3] Ajinomoto Animal Nutrition Europe, 75017 Paris, France; Chalvon-Demersay_Tristan@eli.ajinomoto.com
* Correspondence: dalila.azzout-marniche@agroparistech.fr; Tel.: +33-1-44087244

Received: 1 April 2020; Accepted: 8 May 2020; Published: date

Abstract: Histidine is an essential amino acid (EAA) in mammals, fish, and poultry. We aim to give an overview of the metabolism and physiological effects of histidine in humans and different animal species through a systematic review following the guidelines of PRISMA (Preferred Reporting Items for Systematic Reviews and Meta-Analyses). In humans, dietary histidine may be associated with factors that improve metabolic syndrome and has an effect on ion absorption. In rats, histidine supplementation increases food intake. It also provides neuroprotection at an early stage and could protect against epileptic seizures. In chickens, histidine is particularly important as a limiting factor for carnosine synthesis, which has strong anti-oxidant effects. In fish, dietary histidine may be one of the most important factors in preventing cataracts. In ruminants, histidine is a limiting factor for milk protein synthesis and could be the first limiting AA for growth. In excess, histidine supplementation can be responsible for eating and memory disorders in humans and can induce growth retardation and metabolic dysfunction in most species. To conclude, the requirements for histidine, like for other EAA, have been derived from growth and AA composition in tissues and also have specific metabolic roles depending on species and dietary levels.

Keywords: histidine; metabolism; physiological effects; human; animal species

1. Introduction

Histidine is an essential amino acid in mammals, fish and poultry because it is not de novo synthesized and must be obtained through the diet [1,2]. In these organisms, histidine deficiency induces a decrease in body weight. Moreover, nitrogen balances cannot be maintained with a histidine-devoid diet. In the study by Kriengsinyos et al. [3], it has been shown that histidine deficiency may not affect nitrogen balance if the total protein intake is higher than the current recommendation (0.75 g/kg). However, histidine deficiency induces a decrease in AA oxidation and a decrease in protein turnover. Taking into account these other parameters of protein metabolism, histidine is considered as an indispensable AA in healthy adults. Some studies have also reported the indispensability of histidine for ruminants [4]. Histidine is abundant in red meat and fish, but its content varies among fish species, from histidine-rich fish (dark muscle fish) to histidine-poor fish (white muscle fish). Histidine is present in the body of different species in different forms, including free L-histidine, N (alpha)-acetylhistidine (NAH), histidine-containing protein, and histidine-containing dipeptides such as carnosine and anserine (HRC).

Depending on the species, the requirements and toxicity of histidine differ, as does its effect on metabolism. Therefore, our aim is to present a systematic review following the guidelines of PRISMA [5] (methodology in supplementary material, table S1) of the metabolic and physiological effects of histidine in humans and different animal species. The scope of this review is to compare histidine supplementation on different species, the effect of histidine supplementation on the metabolism, and to analyze the requirement and toxicity for each species. Firstly, common physiological roles of histidine in different species are described, and then, a species-by-species description is made for humans, rodents, pigs, chickens, fish, and ruminants.

2. Common Physiological Roles of Histidine in Different Species

2.1. Histidine Requirements and Metabolism

Histidine requirements are expressed differently among species. For humans, histidine requirements are expressed as daily intake per kilogram body weight. For other species, lysine is frequently used as the reference AA, being known as the first limiting AA. Hence, the requirement patterns for other AAs, including histidine, are expressed in ratio to lysine requirements. According to the Food and Agriculture Organization of the United Nations (FAO), the histidine ratio (percent histidine/lysine) varies: 36 for pigs (20 to 50 kg), 32 for chicks (0 to 3 wk), 36 for rats, 33 for salmonids, 39 for trout, 37 for common carp and 34 for tilapia.

Histidine undergoes different metabolic pathways (Figure 1). It can be methylated to either 1-methyl or 3-methyl histidine, or converted to imidazole-pyruvic acid by transaminase, which produces imidazole-lactic acid by reduction. Transaminase activity is elevated in fetal rat livers and decreases after birth. Its activity can be modulated by diet: it increases when diet protein content is above 25%, whereas it is not modified below 25%. Histidine can be condensed with β-alanine to form carnosine and anserine, which are involved in protection against oxidative stress [6] . It can also be condensed with decarboxylation to form histamine, or it can undergo irreversible degradation. An irreversible, non-oxidative deamination leads to the formation of trans-urocanic acid and ammonia by the histidine ammonia-lyase, also called histidase, which is a cytoplasmic enzyme [6]. To do this, the α-amino group of L-histidine is removed [7]. This is the primary step in histidine degradation that has been studied in mammals and bacteria.

Urocanic acid has a protective role against harmful effects of ultraviolet rays on the skin. It is metabolized in the liver to 4, 5-dihydri-4-oxo-5-imidazolepropanoic acid by urocanate hydratase, and is then converted to formimino-glutamate (FIGLU) and N^5-formimino-tetrahydro-folate. In epiderma, urocanate hydratase is not present, and urocanic acid is the last compound of histidine catabolism. This compound accumulates in the epidermis and acts as a natural sunscreen that protects the skin from the effects of UV light [8,9]. Histidase, located only in the liver and epidermis, is the rate-limiting enzyme of histidine degradation [10]. Its activity is very low in the fetal liver and increases progressively during the first three weeks after birth [11]. Some cases of histidase deficiency have been described. In this context, the main step in histidine catabolism is transamination with the accumulation of imidazole-pyruvate, imidazole-lactate and imidazole-acetate [11]. Gene expression of histidase can be modulated by diet. High protein diets or amino acid-imbalanced diets stimulate its expression [12]. Histidase activity increases proportionally to the increase of protein content in the diet. Rats fed histidine-imbalanced diets have shown a lower food intake and weight gain with a higher histidase gene expression [12]. In the case of undernutrition, there is a decrease in histidase activity and histidase mRNA abundance in the liver. This decrease seems to prevent histidine catabolism [13]. It was also shown that in rats on a low protein diet, a load of histidine induced a lower histidine oxidation compared to rats on an adequate diet [14]. Lastly, some studies have shown that histidase can be controlled by estrogen. In

female rats, the activity of this enzyme is down-regulated upon ovariectomy [15].

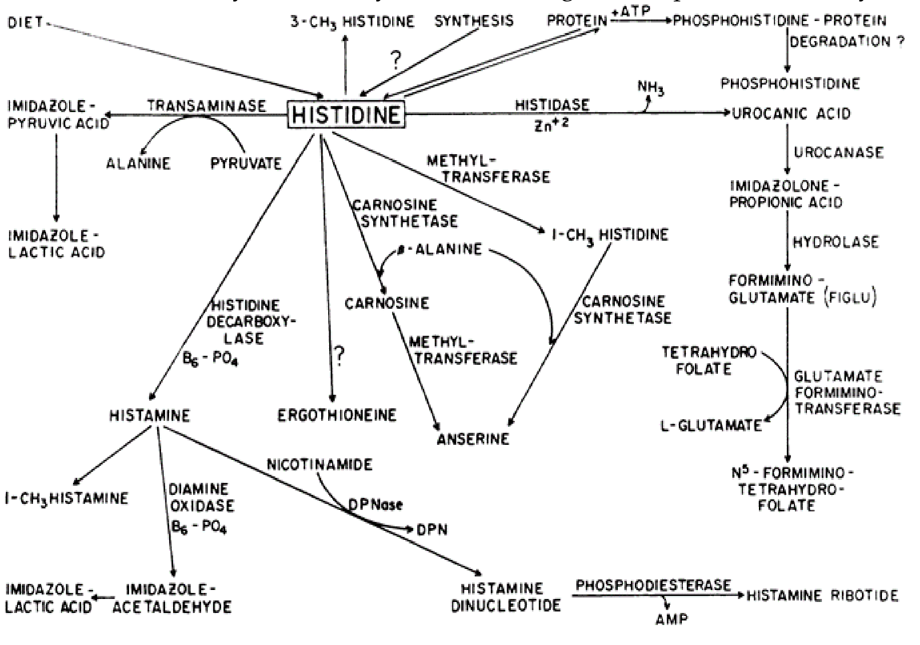

Figure 1. Catabolic pathways of histidine [11].

2.2. Physiological Role of Histidine is Common for all Species

Incorporation into protein: Borsook and Wolf reported that the main region for histidine retention was the viscera. This was determined by the injection of labeled AAs into rodents and the subsequent analysis of the rate of incorporation into protein tissue [16,17]. The maximum rate of histidine incorporation into proteins is slower (max at 30 min after injection of labeled AAs) than for leucine and lysine (max at 10 min for lysine and 20 min for leucine). In guinea pigs, 30 min after intravenous injection of 16 mg/kg of histidine, the rate of incorporation of histidine is 0.9 μM in small intestine and 1.7 μM in liver per g of protein per hour. The highest incorporation was measured in microsome fractions [16].

Synthesis of carnosine and anserine: Carnosine is highly concentrated in muscle (except in cardiac muscle) and brain tissues of all mammals and birds, with a particularly high content in the horse [18]. Anserine is found in skeletal muscle of certain mammals such as rabbits, rats and whales, but is not found in any human tissues [11]. Carnosine has been proven to scavenge reactive oxygen species (ROS) as well as alpha-beta unsaturated aldehydes formed from peroxidation of cell membrane fatty acids during oxidative stress. Carnosine and anserine have a beneficial role as anti-oxidant, antiglycation molecules and anti-aging agents and are furthermore considered functional, bioactive compounds when consumed by humans [19].

Decarboxylation of histidine to histamine by histidine decarboxylase: This metabolic conversion is common in all animal species except fish. This enzyme is present in microorganisms of the large intestine and in many tissues (lung, liver, muscle and gastric mucosa). In contrast to histamine, histidine can cross the blood brain barrier. After crossing the blood-brain barrier, and once in the tuberomammilary nucleus (TMN) of the hypothalamus, histidine is converted into histamine by histidine decarboxylase [20]. Subsequently, histamine is oxidized to imidazoleacetaldehyde and then to imidazoleacetic acid [11]. Histamine is a major mediator in allergic diseases and has multiple effects that are mediated by specific surface receptors on target cells. Inflammation is known as the main pathophysiological characteristic of allergies [21]. Moreover, histamine exerts different effects on inflammation [22]. On the one hand, histamine can induce inflammation contributing to pulmonary fibrosis, cardiovascular diseases and atherosclerosis, atopic dermatitis, colitis, nerve tissue damage, and by influencing Th1/Th2 balance. On the other hand, histamine can regulate inflammation. It is involved in wound healing in skin lesions, in the reduction of colitis severity and

autoimmune encephalomyelitis in experimental models, inhibits tumor development, and microbiota-derived histamine is associated with a reduction of asthma severity [22].

Histamine is a neurotransmitter [23] that plays a role in the control of food intake, along with other neurotransmitters (catecholamines, 5-hydroxytryptamine). Studies have shown that histidine decreases food intake through histamine [20,21]. Vaziri et al. studied the effect of histidine administered by intragastric or intraperitoneal routes to rats and measured food intake after blocking the conversion of histidine to histamine by FMH, a-fluoromethylhistidine. They found that histidine decreased food intake, but that the use of FMH reversed the effect of histidine on food intake [24]. Histamine is also involved in sleep mechanisms [25]. There are histamine-expressing neurons in the TMN that are considered wake-active, owing to histamine that acts via H1 and H3 receptors [26]. Some studies have demonstrated that antihistamine treatment induced sleep, blocking effects of histamine on TMN H1 and cholinergic receptors [27,28].

Buffering role of histidine and histidine-related compounds (HRC*)*: Histidine and HRC have a specific role in the intracellular, non-bicarbonate buffering capacity of vertebrate muscle, which is mainly supported by the imidazole groups of free histidine or histidine residues in proteins as well as HRC, in the pH range of 6.5–7.5 (Figure 2). This metabolic process reduces the decrease of intracellular pH caused by proton accumulation during high-intensity anaerobic exercise, and a consequent ATP hydrolysis to ADP. The decrease of intracellular pH inhibits glycolysis and the buffering capacity of histidine, and HRC enhance the capability for anaerobic exercise performance by stabilizing the pH. The contribution of HRC to buffering capacity among species is described in [29].

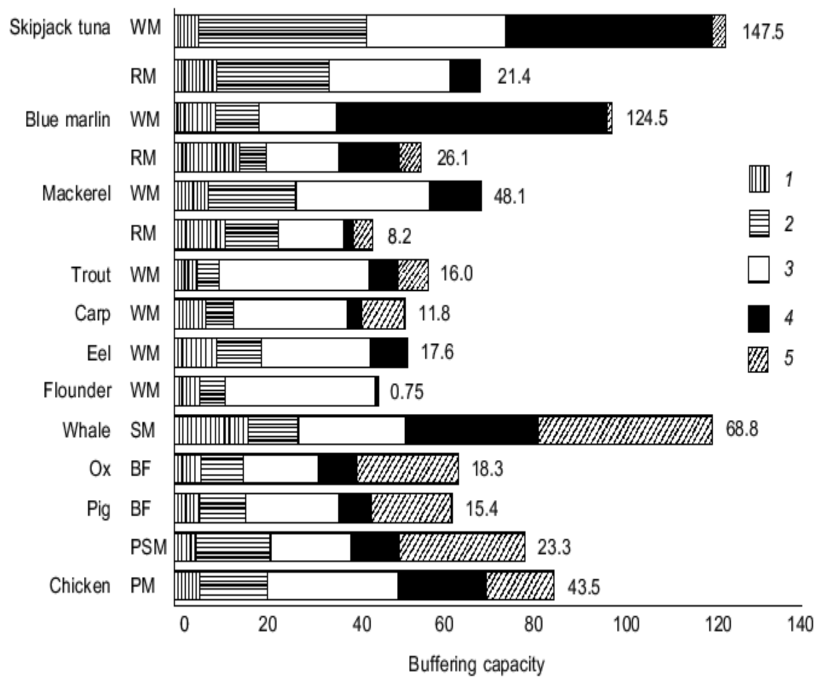

Figure 2. Muscle histidine and histidine-related compounds buffering contribution (4) in comparison with proteins among species (contractile (1) and soluble (2)), inorganic orthophosphate (3), and unknown compounds (5)). Buffering capacity is expressed as μmol NaOH per pH unit per g muscle over pH 6.5–7.5. Values indicated on the bars represent the concentration of total histidine-related compounds (μmol/g muscle). WM, white muscle; RM, red muscle; SM, skeletal muscle; BF, *biceps femoris*; PSM, psoas muscle; PM, *pectoralis minor* [30].

2.3. Histidine Strongly Binds Metal (CO(II), Ni(II), Cd(II), Zn(II), Cu(II), and Fe(III) ions)

The imidazole sidechain of histidine is a common coordinating ligand in metalloproteins. Histidyl residues of metal-activated enzymes and metalloproteins are believed to play an important

role in metal binding by these proteins [31]. Histidine acts on metal regulation, chelating different metal ions such as cobalt(II), nickel(II), copper(II), zinc(II) cadmium(II), and iron(II) [32].

3. Effect of Histidine Level and Supplementation in Humans

According to the FAO, the daily requirement for histidine is 8 to 12 mg/kg of body weight per day in adults. The average intake of histidine in typical, adult diets in Europe, the USA and Japan was reported to be between 2.12 and 2.40 g per day (i.e., about 30 to 35 mg/kg of body weight per day), where men of 50–70 years of age at 5.20 g/day represented the 99th percentile intake [33,34].

3.1. Histidine Intake and Consequences on Body Histidine Status

Steele and Le Bovit [35] have studied histidine tolerance in women who received two levels of L-histidine, 1.5 and 5 g/day. Results showed that at thirty minutes after ingestion, the 1.5 g dose elevated plasma histidine 2- to 8-fold more than the fasting level, while the 5 g dose elevated the level 7- to 11-fold. In both cases (1.5 and 5 g/day), the plasma histidine content remained elevated for a short time and then declined until reaching the fasting level. The rate of fall of the red blood cell histidine was much slower than that of plasma. Block and colleagues [36] also studied histidine tolerance in three healthy adults (two females and one male) that received 5 g of free histidine dissolved in 100 ml water. Blood samples were collected at one, two, and four-hour intervals. One hour after ingestion of 5 g histidine, the amount of histidine in the plasma increased to approximately 7.5, 12.5 and 15 times the fasting level. At the two-hour interval, the amount of plasma histidine for all subjects was less than for the one-hour sample, and continued to decrease. However, it did not return to the fasting level by the end of the tolerance test. These two studies have shown that high doses of histidine rapidly induce a large increase in plasma histidine, that then decrease in a few hours.

3.2. Histidine Supplementation and Eating Disorders

Henkin [37] demonstrated that within four to six days after different amounts of histidine supplementation in healthy, young college men, subjects spontaneously complained of anorexia onset. In volunteers receiving 8 g of histidine daily, anorexia was severe and induced a weight loss of 2–3 lbs over a period of one week, despite persistent encouragement to complete their meals. In general, breakfast was the best-tolerated meal, followed by lunch, and dinner being the least-tolerated meal. With continued administration of larger quantities of histidine, 16 and 32 g daily, all subjects developed a decrease in taste acuity (hypogeusia), and then a decrease in smell acuity, (hyposmia). Further administration of larger doses of histidine was associated with the development of distortions of taste (dysgeusia) and smell perception (dyssomnia). Excessive dietary histidine (50 g of histidine/kg of food) has been reported in some Japanese populations. Okubo and Sasaki [38] examined the correlation between dietary histidine and energy intake among 1689 Japanese female students by using a self-administered diet history survey. Results showed that the ratio of histidine to protein (histidine/protein) was negatively and significantly correlated with energy intake, independent of other dietary factors. Nakajima and colleagues [39] also identified a negative association between histidine/protein and energy intake in 26 male and 38 female students ($r = -0.18$ in men and $r = -0.34$ in women, $p < 0.05$).

In order to investigate the possible efficacy of oral histidine as anorectic therapy in men, Schechter and Prakash [40] fed eight healthy men (ages 32 to 38) with 4 g of histidine supplement per day for four weeks. Results showed that this amount had no significant effect on appetite, taste or smell perception, food intake, or body weight. In addition, total serum zinc, albumin-bound zinc, macroglobulin-bound zinc concentrations, and urinary histidine excretion had not significantly changed. Based on these studies and a risk assessment, the Norwegian Scientific Committee for Food Safety (Holvik et al., 2016) concluded that supplementation with 4 to 4.5 g/day, corresponding to 57 mg/kg of body weight per day for a 70 kg adult, does not have adverse effects in humans. The scientific committee concluded that the specified doses of 0.55 and 0.60 g/day of histidine in food

supplements are unlikely to cause adverse health effects in adults (≥18 years), adolescents (14 to <18 years) or children (10 to <14 years).

3.3. Histidine and Memory Disorders

Geliebter et al. studied the effect of daily histidine doses, from 24 to 64 g, mixed into orange juice, on healthy subjects for four weeks [41]. They reported ensuing headaches, weakness, drowsiness, and nausea in subjects. Two subjects who received 64 g per day reported painful sensations in their eyes and difficult focusing. One subject showed mental confusion after taking 64 g per day, poor memory, and depression with episodes of crying [41]. In contrast, Sasahara and colleagues [42] reported that daily histidine intake (1.65 g/day) actually decreased feelings of fatigue, increased efficiency while performing memory tasks, and promoted clear thinking and concentration in subjects with high fatigue and sleep disruption scores. The large difference in histidine doses likely explains the contradictory effects between studies by Geliebter et al. and Sasahara et al.

3.4. Histidine and Metal Ion Status

Some studies have investigated the effect of histidine on ion absorption. In 113 subjects, for example, iron absorption was not affected by histidine supplementation (416 to 2080 mg/day) [43]. In another study, zinc histidine complexes were better absorbed than zinc sulfate in ten healthy subjects. Therefore, the ingestion of zinc complexes with histidine at a ratio of 1:2 or 1:12 increased serum-zinc concentration 25% more than ingestion of zinc sulfate. No changes in zinc excretion was observed in this study [44]. In 1980, Henkin and colleagues studied the effects of graded increases of histidine doses (8.1–64.8 g, daily) in healthy volunteers and in patients with scleroderma. They observed a graded increase in urinary zinc excretion. In subjects receiving high doses of histidine, urinary zinc excretion increased from 454 ± 50 μg per day to 5269 ± 840 μg per day compared to the control state. In some subjects that received the highest level of histidine supplementation (64.8 g daily), approximately 0.5% of the estimated total body zinc pool was lost through urine each day.

Menkes disease (MD) is a neurodegenerative disorder caused by mutations in the *ATP7A* gene, and can lead to death in early childhood. Symptoms of this disease are attributed to deficient activity of Cu-dependent enzymes (cytochrome oxidase, tyrosinase, superoxide dismutase, dopamine beta hydroxylase, lysyl oxidase, and sulfhydryl oxidase). In patients suffering from MD, copper therapy has been shown to be inefficient. Interestingly, however, due to the chelating effect, copper histidine therapy is beneficial in reversing the skin and hair changes, improving appendicular tone, socio-cognitive milestones, weight gain, and immunity [45–47].

3.5. Histidine and Skin Dysfunction

Histidine status seems to be important in skin dysfunction and various cutaneous diseases. The deficiency in histidine, as in other essential AA (Ile, Leu, Lys, Met, Cys, Phe, Tyr, Thr, Trp, Val, Arg, or Gln) significantly decreased hyaluronan levels in human dermal fibroblasts [48]. Hyaluronan plays an important role in tissue repair and in cellular proliferation and migration in skin. Moreover, Voorhees et al. [49] detected an increasing quantity of labelled histidine within keratohyalin, a "histidine-rich" protein (HRP) that has been isolated and characterized in human necropsy epiderma. However, no changes were observed in HRP synthesis in psoriatic lesions. Regarding dermatitis disease, Tan and colleagues reported that daily oral histidine intake (4 g per day) increased filaggrin formation. Filaggrin is a protein that constitutes the granular layer of keratynocytes that contributes to barrier function through skin hydration and maintenance of stratum corneum acidity. This increase of filaggrin induced a decrease of atopic dermatitis disease severity [50].

3.6. Histidine and Metabolic Syndrome

Dietary histidine may be associated with factors improving metabolic syndrome related to obesity. An internet-based, cross-sectional study conducted in a northern Chinese population by Yan-Chuan Li and colleagues [51] reported an association between higher dietary histidine intake (1400

mg/d), above dietary requirements (8 to 12 mg/kg of body weight per day in adult according to FAO, corresponding from 560 to 840 mg/day for a healthy adult of 70 kg) and lower prevalence of overweight conditions and obesity, lower BMI, waist circumference, and blood pressure. Insulin resistance, which is an important feature of the metabolic syndrome, seems to be improved in overweight and obese individuals with histidine supplementation [51,52].

Obesity is also associated with a high level of pro-inflammatory cytokines and inflammation biomarkers [53]. In the study by Li et al., results show that dietary histidine is inversely associated with some pro-inflammatory cytokines such as TNF-α, IL-1, IL-6, and the inflammation biomarker CRP. These results are consistent with what was found by the team of Lee [54]. It seems that supplementation with histidine might improve inflammation in overweight and obese people. Adiponectin, an adipocytokine that appears to play a role in the development of insulin resistance, could also be involved in histidine's effect on obesity. A positive correlation was also found between histidine supplementation and serum adiponectin in overweight and obese individuals. Histidine could increase secretion of adiponectin by inhibiting oxidative stress and inflammation in adipose tissue [51].

4. Effect of Histidine Level and Supplementation in Rodents (Rats and Mice)

4.1. Effect on Food Intake

Many studies have shown that histidine acts on feeding behavior [55]. Results of these studies in rats reported that food intake is decreased with increased dietary histidine [20,56]. Kasaoka and colleagues showed a decrease in food intake with a daily dose of 20% casein plus 2.5% or 5% histidine (25 g/kg or 50 g/kg of diet) for eight days, compared with a group that received 20% casein without supplemental histidine. The decrease in food intake caused by dietary histidine can be explained by an activation of histamine neurons [55,57]. Intraperitoneal or intracerebroventricular injection of L-histidine also suppresses food intake. In addition, inhibition of histidine decarboxylase (HDC) reverses this effect, which suggests that histidine suppresses food intake through its conversion into histamine in the hypothalamus [58]. The released histamine acts on food intake through histamine H1 receptors [55]. All of these studies have shown that a higher histidine intake induces a decrease in food intake. As in humans, this decrease in food intake seems to be due to the effect of histamine.

In contrast to these studies showing a decrease in food intake, a study by Holeček demonstrated an increase in food intake in rats that drank histidine-enriched water [59]. They hypothesized that in other studies, the histidine-enriched diet induced an imbalance in AA concentration, leading to competition for cellular transporters to the brain. There was then a reduction of several AA in the brain causing the decrease in food intake. While in their study, to balance the increase in histidine in water, rats increased their food intake. The results might be different if it was not the water but the diet that was enriched with histidine.

4.2. Effect on Neuroprotection

Cerebral ischemia is a major cause of death in adults. Six hours after a cerebral ischemia, astrocytes are activated to induce neuron survival [60]. To avoid the spread of the lesion and to induce the local immune response, astrocytes come together to form a barrier called the glial scar. This barrier can inhibit neurogenesis at a late stage after cerebral ischemia [61].

An early study by Liao and colleagues [62] showed that histidine, converted into histamine, seems to induce neuroprotection at an early stage after cerebral ischemia. With its action on neurons, more specifically on astrocytes, histidine protects them from oxygen-glucose deprivation-induced injuries and inflammatory cells by inhibiting their recruitment. More recently, the same researchers demonstrated that there is also an effect on the late stage after cerebral ischemia [63]. A dose- and stage-dependent treatment with histidine at a high dose of 1000 mg/kg at an early stage, and a decreased dose of 500 mg/kg at a late stage induced astrocyte migration toward the infarct area. This migration enables neurogenesis in the lesion area, indicating that histidine provides long-term neuroprotection after cerebral ischemia.

Some studies have reported that histidine could protect against epileptic seizures. A study by Chen et al. [64] showed that seizures induced in rats by pentylenetetrazole, a central nervous system (CNS) stimulant, could be reversed by histidine in a dose-dependent manner. The study reported that with 500 mg/kg of histidine in rats, there was a large increase of histamine that entered the cortex, hippocampus and amygdala. These observed effects seemed due to the increase in histamine synthesis owing to its precursor, histidine. A decrease in histamine led to an increase in the duration of epileptic seizures [65], which suggests that there is a link between seizures and histamine concentration in the brain. Actions of histamine on convulsions could be mediated through presynaptic H3-receptors and postsynaptic H1-receptors. Moreover, one study suggested that anserine, which is water soluble, could be an active peptide. In mice, the anserine supplementation improved memory functions in Alzheimer disease-model mice by exerting a protective effect on the neurovascular units, which are composed of endothelial cells, pericytes, and supporting glial cells. Therefore, histidine could also have a neuroprotection effect through anserine production [66].

4.3. Effect on Growth and Metabolic Dysfunctions

In a study in which the level of histidine was >2 g/kg BW/day, rats from 7 to 46 weeks showed growth retardation, hepatomegaly and hypercholesterolemia [67,68]; when histidine was >4 g/kg BW/day, hypercholesterolemia was observed after 46 days of treatment [69].

An excess of histidine can have a negative impact on growth, but the effect can be mitigated by increasing dietary protein content. Muramatsu et al. [70] studied the adverse effect of excess levels of histidine in growing rats. When male weanling rats were fed with a diet consisting of 10% casein supplemented with 50 g/kg BW/day of histidine for three weeks, growth was reduced by 77%. However, in rats fed a 25% casein diet supplemented with 50 g/kg BW/day of histidine for three weeks, growth decreased by 23% compared to the unsupplemented control rats [71,72]. Another study [73] showed that, at 6% casein and 20 g/kg diet histidine, growth depression was severe. However, when the protein content of the diet was increased to 12%, the degree of growth depression was lessened. This indicates that rats can better tolerate an excess of histidine with an increased protein content in the diet. These findings are consistent with the observation that activities of histidine-catabolizing enzymes are higher in rats fed moderate- and high-protein diets compared to low-protein diets, and that this results in an enhanced ability to degrade excess histidine with a concomitant decrease in histidine toxicity [71,72]. A 13-week feeding study sought to evaluate the effect of diets containing 0, 3.1, 6.2, 12.5, 25, and 50 g/kg of histidine (L-histidine monohyrochloride) on male and female F344 rats [74]. With the higher dose of histidine, body weight and food intake decreased in males and demonstrated an increase in hemoglobin volume and hematocrit. This effect seemed due to the high dose of histidine, because it is a major component of hemoglobin. The level of blood urea nitrogen and creatinine increased in females, and the level of blood urea nitrogen also increased in females fed 12.5 g/kg histidine. Concerning body composition, there was an increase in the weight of some organs, including the kidney for males of the 25 g/kg and 50 g/kg groups, testis for males of the 50 g/kg group and kidney for females of the 50 g/kg group. Owing to the different adverse effects observed for 50 g/kg fed rats, the authors concluded that the maximum tolerable dose of histidine is 25 g/kg of the diet. In obese rats, histidine (1875 g/kg BW) was seen to improve inflammatory and oxidative status [75] via the NF-κB- and PPARγ-involved pathways.

4.4. Effect of Histidine on Mineral Metabolism

As previously described, histidine can chelate metal ions. Some studies have investigated the effect of histidine intake on iron, copper and zinc absorption and retention. Tissue levels of iron and fecal losses of iron were not affected by the histidine supplementation [76]. When histidine was added to an ^{59}Fe solution that contained ascorbic acid, it increased absorption [77]. This suggests some direct reaction between iron and histidine and is consistent with the hypothesis that an AA-iron chelate is formed and subsequently absorbed. Regarding copper, no effect of histidine intake was observed in rats [76]. In vitro, histidine was found to facilitate copper uptake in hepatic, placental, and brain cells [78–81]. Some studies have investigated the effect of histidine-feeding on zinc status, finding that the

effect of histidine supplementation is controversial. Freeman and Taylor [82] *in*vestigated effects of both acute (intravenous infusion of 250 mg/hr histidine) and chronic (500 mg/day was given by gavage for 43 days) histidine supplementation. They reported an increase in zinc excretion by three to six times following acute and chronic administration of histidine. Elsewhere, however, the decrease of plasma zinc has been observed in acute, but not chronic, supplementation. In young adult rats, dietary repletion with zinc chloride supplemented with L-histidine (40 mg/kg of diet) was more effective at reversing cognitive impairment due to zinc depletion, rather than repletion with a zinc salt alone. This effect could be explained by the stimulation of ^{65}Zn transport across the brain endothelium when histidine was added to perfusion [83,84]. Snedeker and Greger [76] reported that zinc absorption and utilization were more strongly impacted by protein levels in the diet than by the level of histidine. Nonetheless, other studies have reported that histidine could induce a zinc deficiency. In rats fed histidine levels superior to 4 g/kg BW/day from seven to 46 weeks, a significant reduction in plasma zinc was observed. Interestingly, supplementation of a rat diet with 50 g/kg histidine did not affect the rate of turnover of zinc (the rate of turnover of ^{65}Zn from two to four weeks after a single injection of the tracer), whereas the diet supplemented with 8% histidine induced a severe zinc deficiency (50% reduction in the plasma zinc content) [85]. This effect of histidine supplementation on zinc status could depend on dietary zinc intake. Indeed, when rats were fed a zinc-adequate diet, histidine supplementation did not cause any changes to the zinc status (zinc concentrations, ^{65}Zn tissue distribution, and tissue-specific activities). However, when zinc intake was low, histidine supplementation led to a lower ^{65}Zn retention, associated with increased fecal excretion and a shorter biological half-life [86].

4.4. Histidine and Cancer

Recent data suggest that histidine catabolism and intake influence the sensitivity of cancer cells to methotrexate [87]. Methotrexate is an anticancer treatment for certain solid tumors and blood cancers, but may be toxic for various non-cancer cells. CRISPR/Cas9-mediated depletion of formimidoyltransferase cyclodeaminase (FTCD), histidine ammonia lyase (*HAL*), and amidohydrolase domain containing 1 (*AMDHD1*) decreased the sensitivity of cells to methotrexate. Moreover, methotrexate and histidine treatment of mice induced a marked decrease in tumor size that was significantly greater than in any of the other treatment cohorts. Taken together, these data highlight the role of the histidine degradation pathway in the efficiency of chemotherapy agent methotrexate [88].

4.5. Toxicity Dose

Toxicity doses have been studied in rats from different perspectives: their carcinogenicty as well as lethal doses. No mortality or adverse effects were observed during a 13-week period during which rats were fed with different amounts of histidine (containing 0, 3.1, 6.2, 12.5, 25, and 50 g/kg of histidine) [74]. Another study investigated the long-term toxicity and carcinogenicity of histidine in 100 rats for 104 weeks [89]. This study was performed using two groups, 50 males and 50 females, fed with diets containing 0, 12.5, and 25 g/kg histidine. They observed that tumors developed in all three groups. However, these tumors were the same as those that develop spontaneously in F344 rats. No significant differences for the incidence or the type of tumor were observed between these three groups. The authors concluded that histidine was not carcinogenic in these rats under their experimental conditions. Gullino and colleagues [90] studied toxicity of AAs. They studied the DL50, the dose of histidine that kills 50% of rats, and the DL99.9, the dose that kills all rats. The toxicity data showed that the DL50 was 23 mmol/kg of body weight and the DL100 was 33 mmol/kg of body weight. There was no difference concerning the dose between the two isomers L-histidine and D-histidine.

5. Effect of Histidine Level and Supplementation in Breeding Farmed, Monogastric Pigs and Chickens

5.1. Histidine in Pigs

Figueroa and colleagues [91] studied the effects of AA supplementation on growing pigs submitted to different levels of crude protein (CP) (16%, 12%, or 11% CP), where the limiting AA CP diet (11%) was supplemented or not with histidine or other AAs. The diet at 12% of CP and 11% of CP induced a reduction of growth performance, even if the diet was supplemented with lysine, tryptophan, threonine, and methionine. Results showed that the performance was higher with 16% or 12% CP compared to 11% CP, and that histidine supplementation alone failed to reverse this effect. However, when the supplementation of histidine was combined with valine, pigs exhibited similar feed intake and weight gain compared to the pigs fed the 16% or 12% CP diet, suggesting that valine is limiting before histidine in the diet. Of note, gain-to-feed, defined as the ratio of body weight gain to feed consumption, was still significantly higher with pigs fed the 16% CP diet compared to 11% CP. Lastly, results showed a large reduction in plasma urea concentrations correlated to the decrease in crude protein level in the diet. Supplementation with histidine with valine or valine + isoleucine further reduced plasma urea at d14. Some studies have investigated the effect of dietary supplementation of blood meal as a source of histidine and its impact on anserine and carnosine synthesis. A diet of 95% basal diet +5% blood meal tends to increase the concentration of carnosine in two types of muscle, longissimus dorsi and vastus intermediate [92]. However, this is not the case for anserine, probably due to the little activity of carnosine methylation compared to anserine in pig muscles. Dietary supplementation of blood meal by increasing the histidine concentration seems to be a useful diet strategy in pigs to produce carnosine-enriched pork, as observed in broiler chickens.

5.2. Histidine in Chicken

Few studies have investigated the effect of histidine supplementation in chicken. High supplementation of histidine (4 g/kg on an as fed basis) was shown to reduce average daily gain by 48 to 51% in eight-day-old crossbred chicks [93]. Similarly, growth depression of 31% was observed with a diet supplemented with 3 g/kg of histidine. The main histidine role described for chickens is that of an anti-oxidant through carnosine and anserine, which have a significant anti-oxidative activity. Auh et al. and Park et al. [94,95] have shown that diet supplementation with 5% blood meal increases the concentration of carnosine in chicken breast. In connection with this property, various studies have evaluated diet supplementation with histidine to increase the anti-oxidant capacity of muscle and the nutritional value of meat. A study by Kralik et al. [96] showed that supplementation with histidine induced an increase of carnosine concentration in breast muscle tissue. Diet supplementation with 0.3% histidine induced an increase of carnosine by 8.88%, and a supplementation with 0.5% histidine increased carnosine concentration by 25.96%. Haug et al. [97] experimented with different diets supplemented with histidine, at 1 g/kg, 2 g/kg, and 3 g/kg. They found that with a supplementation of 1 g/kg, there was an increase in breast muscle carnosine concentration of 62% compared to the control group. Kai et al. [98] examined the carnosine and anserine content in muscles of female broilers fed with diets supplemented with graded levels of histidine at 67%, 100% (control) and 200% of histidine requirements according to the NRC (1994). They reported that these dipeptides were decreased in the low histidine group (67%). In particular, carnosine was not detected in muscles in this group. In contrast, these two dipeptides were increased in the high histidine group (200%).

Kopec studied the effects of histidine and zinc supplementation on broiler chickens [99]. To do so, they used supplementation with histidine (6.83 g/kg diet), either using the pure AA or spray-dried blood cells (SDBC) rich in histidine (6.14 g/kg diet), with or without Zn. There was an increase in anserine/carnosine content in muscle with the pure AA and with SDBC. Concerning enzyme activity, supplementation of histidine enables an increase in superoxide dismutase activity in muscles, and erythrocytes and glutathione peroxidase activity in plasma, compared to the control diet without histidine supplementation.

In conclusion, the addition of histidine in the diet can increase carnosine and anserine and improve meat quality.

6. Effect of Histidine Level and Supplementation in Fish

6.1. Histidine Effect on Fish Growth

In grass carp fed a histidine-supplemented diet for two weeks (2.0 [control], 3.7, 5.9, 7.9, 9.8 and 12.2 g/kg diet), the dietary histidine requirement of young grass carp (279.1–685.4 g) was 7.63 g/kg, based on growth performance [100]. Above this level, growth performance was lower (Figure 3).

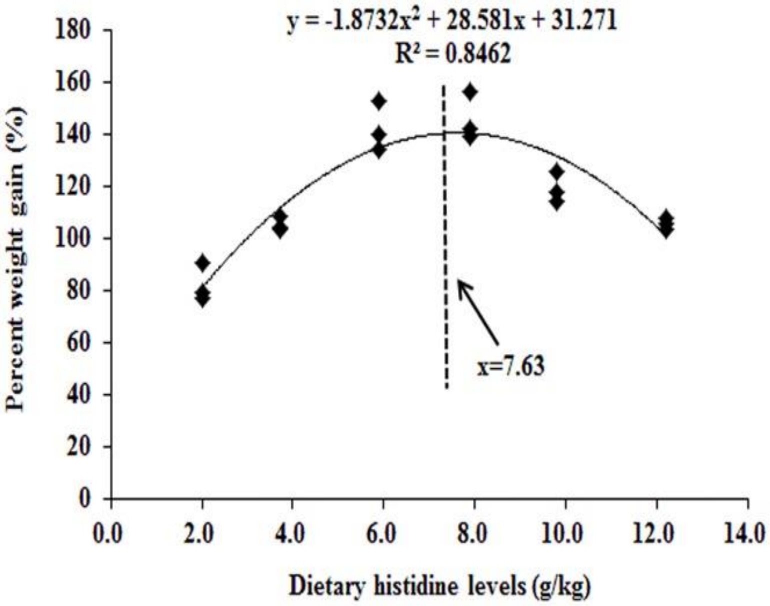

Figure 3. Quadratic regression of the weight gain percentage of grass carp as a function of histidine dose [100].

In catfish, the requirement was demonstrated to be 3.7 +/−0.1 g/kg of histidine based on dry diet, or 15.4 g/kg of histidine when expressed as a percentage of dietary protein. Dietary histidine caused no increase in serum-free histidine levels until the dietary requirement was reached. Muscle carnosine could not be detected in the catfish [101]. In Nile tilapia juveniles fed diets containing graded levels of histidine (4.2, 5.4, 7.1, 8.9, 9.8, and 11.5 g/ kg dry diet), the final weight, weight gain, feed conversion ratio, protein efficiency ratio and net protein utilization were optimized in fish fed 8.9 g/kg of histidine kg. However, whole-body protein content was higher in fish fed 7.1 and 9.8 g/kg of histidine kg dry diet. Contrary to mammals and birds, fish are able to recruit new muscles fibers throughout their lifetime. Muscle growth can be modulated by different molecules as myogenic regulatory factors. This study showed that myogenin expression was higher in fish fed 9.8 and 11.5 g/kg of histidine kg dry diet, compared to fish fed 4.2 to 7.1 g/kg of histidine kg dry diet [102]. These results demonstrated that dietary histidine intake positively affects muscle growth.

6.2. Buffering Role of Histidine in Fish

Fish contain a large amount of free histidine and HRC. The HRC buffering capacity has been studied by Abe and al. [103] in two fish species: rainbow trout (*Salmo gairdneri*) and the Pacific blue marlin (*Makaira nigricans*). They found that HRC contribution to total cellular buffering varied from a high of 62% for white marlin muscle to a low of 7% for red trout muscle. These results suggest that the HRC buffering capacity depends on fish species and type of muscle. In red muscle, other principal buffers are phosphate and protein with taurine. Buffering capacities of histidine could act through N (alpha)-acetylhistidine (NAH) presence in very high concentrations exclusively in the brain and lens of ectothermic vertebrates, including ray-finned fishes, amphibians and reptiles, but not in those of endothermic birds and mammals. Although NAH is known to be synthesized from histidine and acetyl-CoA by histidine *N*-acetyltransferase (HISAT; EC 2.3.1.33), the gene that encodes HISAT remains unidentified. It was observed that 13 species (seven cichlids, five anabantids, and one catfish)

contained considerable amounts of NAH in their skeletal muscles (>1 μmol/g). The highest level of NAH (10.37 μmol/g) was found in the tissue of *Betta splendens* (Siamese fighting fish). Moreover, the NAH contents in the tissues of *Trichogaster trichopterus* (three spot gourami), *Kryptopterus bicirrhis* (glass catfish), *Oreochromis niloticus* (Nile tilapia), *Mikrogeophagus ramirezi* (ram cichlid), and *Parachromis managuensis* (Guapote tiger) were 3.17–-6.16 micromol/g. The skeletal muscle of amphibians (five species) and reptiles (four species) had a low level (< 0.25 μmol/g) of NAH [29].

6.3. Role of Histidine in Preventing Cataracts in Fish

Another functional aspect of histidine is its capacity to decrease the incidence and severity of cataracts in fish. Cataracts are a major concern in the aquaculture industry. This function of histidine was suggested after an outbreak of cataracts in rapid growth Atlantic salmon fed with feeds free of blood meal, a good source of histidine in the diet [104].

N (alpha)-acetylhistidine (NAH) is a prominent biomolecule in the lens; lens NAH concentrations directly reflect dietary histidine levels in Atlantic salmon. Dietary histidine appears to be one of the important factors in preventing cataracts. The beneficial effects are related to high levels of histidine and the buildup of NAH in the lens, which possess buffering and antioxidant properties [105]. In cultured lenses, the concentration of NAH was significantly reduced in those that were oxidatively stressed. Also, the innate antioxidative defense system was influenced by histidine enrichment in the media through an increase of glutaredoxin expression [106]. NAH in lens has moreover been reported to be involved in lens-water homeostasis. The volume homeostasis and lens transparency were reported to be particularly important in both freshwater and seawater. NAH is exported to ocular fluid where a specific acylase cleaves histidine, which is then actively taken up by the lens and re-synthesized into NAH. Each NAH molecule released into ocular fluid down its gradient carries 33 molecules of bound water, thus effectively transporting the water against a water gradient. Therefore, NAH functions as a molecular water pump to maintain a highly dehydrated lens and thus prevents cataract formation [107]. Dietary levels of histidine modulate lens NAH concentration and have been found to prevent or slow the progression of cataract development. Different studies have investigated the effect of dietary histidine on cataract severity and prevalence. Trösse et al. [108], for instance, reported that lens N-acetyl histidine contents reflected dietary histidine levels. They found that NAH contents were negatively correlated to cataract scores in adult Atlantic salmon (1.7 kg) fed three different amounts of dietary histidine (9, 13, and 17 g histidine/kg diet) for four months. Microarray analysis of the lens transcriptome revealed that among the differentially expressed transcripts were metallothionein A and B (1.5–1.7), as well as transcripts involved in lipid metabolism (Fatty acid-binding protein. Intestinal -2.1), carbohydrate metabolism, regulation of ion homeostasis, and protein degradation. Similarly, in salmon smolts fed different levels of histidine (10, 12, 14, 16 and 18 g histidine/kg diet) for 13 weeks, lens NAH concentrations were positively correlated with histidine intake. Furthermore, cataract prevalence and severity were negatively correlated with the dietary histidine concentration. The authors concluded that the amount of dietary histidine that minimized the risk of cataract development was 14.4 g histidine/kg of diet [107]. In an interesting experiment, Waagbø et al. [109] investigated the effects of histidine diet content (9.3, 12.8 and 17.2 g/kg of diet) and feeding time period (June to July, July to September, September to October) on cataract severity. They observed the development of severe cataracts between July and September [109]. The cataract severity was directly related to the dietary histidine level fed during the first and second periods. The authors concluded that the risk of cataract development could be prevented by histidine supplementation just before and during the early phase of cataract development.

7. Effect of Histidine Level and Supplementation in Ruminants

7.1. Effect of Histidine on Growth

Schoof et al. [110] explored whether histidine is the first limiting AA for growing ruminants. As such, they used a canula to evaluate the effects of continuous duodenal infusion of histidine on the

retention of nitrogen and AA (AA) utilization. They conducted three experiments. In experiments I and II, young bulls were fed with a basal diet containing 125 g of crude protein/kg dry matter, infused intraduodenally with 8 g histidine per day. In experiment III, growing bulls were fed with a low-protein diet containing 94 g CP/kg dry matter, infused with 6 g of histidine per day. Results showed that with the normal-protein or low-protein diet, there were no significant differences for N retention between treatments with or without histidine infusion. The utilization of individual AA was calculated with the ratio 'retained AA to apparently digested intestinal AA'. They found that the most used AA was histidine at 80%, followed by arginine (72%), methionine (60%), leucine (45%), and lysine (44%) during periods without supplementation of histidine. The study concluded that histidine from the basal diet was sufficient to maintain growth under the experimental conditions employed. Nevertheless, in all AA present in the duodenum, histidine could be the first limiting AA for growth [110]. Similarly, a study by Oldham [111] also concluded that histidine was the first limiting AA in raising cattle with maize silage.

On the other hand, a study by Gabel and Poppe [112] used a factorial method to demonstrate that the quantity of histidine present in the rumen and from the diet was insufficient to maintain growth in young bulls.

7.2. Effect of Histidine on Milk Protein Synthesis

Gao and colleagues studied the effects of leucine and histidine, separately, on the mTOR signaling pathway in milk protein synthesis [113]. To do so, they used CMEC-H (bovine mammary epithelial cells) in Earle's balanced salt solution (EBSS), devoid of AAs to eliminate the impact of other AAs, and supplemented with Leucine (Leu) or histidine. Results showed that Leu and histidine stimulated the expression of different casein forms: αs-casein, β-casein, and κ-casein. This suggested that Leu and histidine are limiting factors for milk protein synthesis. Subsequently, the authors studied the effects of histidine on molecules involved in protein synthesis. It is well known that mTOR is a key regulator of milk protein synthesis, and it seems that AAs play a role in mTOR regulation due to a phosphorylation on Ser^{2448}. This study proved that there is another phosphorylation site, Ser^{2481}, that is an indicator of mTOR pathway activation in CMEC-H. P-mTOR was increased with addition of histidine in the full concentration range (0.15, 1.20, 4.80, and 9.60 mmol/L). They also studied the effects of histidine on Raptor and GβL, two other mTORC1 components. Their expression was also increased with histidine supplementation compared to the control group. Concerning S6K1, an mTOR target, there was an increase in phosphorylation within all concentration ranges. This result contrasts to what was found by Prizant and Barash [114], but is in accordance with the study by Toerien et al. [115]. In the study by Prizant and Barash, CMEC (bovine mammary epithelial cells) were incubated with histidine for ten minutes, while in Gao's work, they were incubated for six hours. Gao and al suggest that the lack of AAs in the medium induced a stress and the supplementation of histidine improve cell survival before milk protein synthesis. There was also an increase in P-4EBP1 with histidine supplementation, which is in accordance with the fact that mTOR regulates protein translation by inducing the phosphorylation of 4EBP1. Gao's work seems to show that with the addition of histidine, there is an increase in casein expression through activation of the mTOR pathway.

Another study investigated the effects of histidine-supplemented drinking water on the performance of lactating dairy cows [116]. Their hypothesis was that a sufficient proportion of histidine added to drinking water would have an effect on milk synthesis in lactating cows. Eight dairy cows were fed with an ad libitum corn and alfalfa silage-based, total mixed ration. The cows had access to water enriched with 0 or 2.5 g/L of histidine, in a crossover design of two periods of 7 days. When histidine was added to the drinking water, water intake increased from 85.1 to 92.1 L/d. On the last day of each period, plasma samples were collected: the concentration of histidine was increased from 14.6 to 21.6 μM, while the concentration of other AAs was not affected. Moreover, the supplementation with histidine had an impact on milk yield with an increase of 1.7 L/d, and on lactose yield with an increase of 90 g/d. This was accompanied by an increase in protein yield and a decrease in fat percentage. However, the effect of water intake was not discussed.

S. Hadrova et al. [117] evaluated the effect of histidine on milk yield and milk composition on high-yield, lactating Holstein cows. The cows were fed with a histidine deficient diet (18% deficiency) and with a diet supplemented in histidine at 13.6 g/d. The food intake did not differ between these two treatments. In accordance with a study by Doelman et al. [116], the yields of milk, protein, casein and lactose were significantly higher with histidine supplementation compared to the control group. Their results seem to show that histidine could be a limiting AA for lactation in dairy cows, in cases where the diet is composed of methionine, lysine, and leucine in proportions that meet requirements.

8. Conclusion

Histidine is an essential AA and its requirements have been derived from growth and AA composition in tissues. In addition to its role in protein metabolism, histidine, as a functional AA, has specific metabolic roles.

As such, when histidine is limiting in the diet in relation to the need for growth, its supplementation can be associated with a better performance. When this supplementation provides histidine beyond growth requirement, other benefits can be observed as described exhaustively in this paper. However, when this supplementation provides histidine in excess, AA imbalance can trigger a decrease in growth and food intake and other unwanted effects. These dose-effects of histidine are summarized thereafter:

- In human, the daily requirement for histidine is 8 to 12 mg/kg of body weight per day in adults, and the average intake in typical adult diets in Europe, USA and Japan was reported to be between 30 to 35 mg/kg of body weight per day (2.12 and 2.40 g per day). Daily histidine supplementation, under 2 g/daily, improves inflammation in overweight and obese people, decreases feelings of fatigue, and increases concentration and efficiency at work. A daily intake of 4 g decreases severity in atopic dermatitis disease. If the supplementation increases up to 8 g of histidine, a severe anorexia is induced. Finally, supplementation with a large dose, from 16 to 64 g/day, induces a decrease in taste and smell acuity, and the onset of headaches, weakness, drowsiness, nausea, and memory disorder.
- In rodents, histidine supplementation up to 25 g/kg of diet is beneficial. A large dose of 50 g/kg of diet can have different adverse effects, such as a large decrease in body weight gain, in food intake, and a growth retardation. Due to this negative effect, maximal tolerable dose of histidine was fixed to 25 g/kg of the diet.
- In pigs, histidine, when combined with other amino acids, can improve performance and amino acid balance in a context of low-protein diet
- In chickens, the major benefit of histidine supplementation is the increase in carnosine and anserine in the muscle allowing an increase in anti-oxidant capacity.
- In salmonids, a beneficial effect of histidine supplementation is its capacity to decrease the incidence and severity of cataracts.
- In ruminants, histidine supplementation induces an increase in milk yield and an increase in milk protein synthesis.

To conclude, histidine supplementation can be interesting for different aspects depending on the species. In humans, data suggest both clinical and nutritional interests of histidine supplementation as a strategy to improve metabolic syndrome features, skin dysfunctions, and memory. In animal production, histidine supplementation can be of interest to promote both the yield and quality of end-products. The indispensability of histidine, and the accumulating evidence of its importance in numerous physiological functions make histidine a unique AA which deserves further consideration in both human and animal nutrition.

Supplementary Materials: The following are available online at www.mdpi.com/xxx/s1, Table S1: The PRISMA Statement.

Author Contributions: Conceptualization, T.C-D.; D.T.; D.A.-M. Writing—original draft preparation, J.M and D.A.-M.; writing—review and editing, J.M., D.A.-M., D.T., P.S., T.C.-D.; All authors have read and agreed to the published version of the manuscript.

Funding: Supported by Ajinomoto.

Conflicts of Interest: None of the authors has any conflict of interest

References

1. Rose, W.C. The amino acid requirements of adult man. *Nutr. Abstr. Rev.* **1957**, *27*, 631–647.
2. Wu, G. Amino acids: Metabolism, functions, and nutrition. *Amino Acids* **2009**, *37*, 1–17, doi:10.1007/s00726-009-0269-0.
3. Kriengsinyos, W.; Rafii, M.; Wykes, L.J.; Ball, R.O.; Pencharz, P.B. Long-term effects of histidine depletion on whole-body protein metabolism in healthy adults. *J. Nutr.* **2002**, *132*, 3340–3348, doi:10.1093/jn/132.11.3340.
4. Onodera, R. Essentiality of histidine in ruminant and other animals including human Beings. *Asian-Australas. J. Anim. Sci.* **2003**, *16*, 445–454, doi:10.5713/ajas.2003.445.
5. Moher, D.; Liberati, A.; Tetzlaff, J.; Altman, D.G.; PRISMA group preferred reporting items for systematic reviews and meta-analyses: The PRISMA statement. *PLoS Med.* **2009**, *6*, e1000097, doi:10.1371/journal.pmed.1000097.
6. Taylor, R.G.; Levy, H.L.; McInnes, R.R. Histidase and histidinemia. Clinical and molecular considerations. *Mol. Biol. Med.* **1991**, *8*, 101–116.
7. Mehler, A.H.; Tabor, H. Deamination of histidine to form urocanic acid in liver. *J. Biol. Chem.* **1953**, *201*, 775–784.
8. Kinuta, M.; Ubuka, T.; Yao, W.B.; Zhao, Y.Q.; Shimizu, H. Isolation of S-[2-carboxy-1-(1H-imidazol-4-yl)ethyl]-3-thiolactic acid, a new metabolite of histidine, from normal human urine and its formation from S-[2-carboxy-1-(1H-imidazol-4-yl)ethyl]cysteine. *Biochem. J.* **1994**, *297*, 475–478, doi:10.1042/bj2970475.
9. Hais, I.M.; Štrych, A. Increase in urocanic acid concentration in human epidermis following insolation. *Collect. Czechoslov. Chem. Commun.* **1969**, *34*, 649–655, doi:10.1135/cccc19690649.
10. Zannoni, V.G.; La Du, B.N. Determination of histidine α-deaminase in human stratum corneum and its absence in histidinaemia. *Biochem. J.* **1963**, *88*, 160–162.
11. Stifel, F.B.; Herman, R.H. Histidine metabolism. *Am. J. Clin. Nutr.* **1971**, *24*, 207–217.
12. Torres, N.; Beristain, L.; Bourges, H.; Tovar, A.R. Histidine-imbalanced diets stimulate hepatic histidase gene expression in rats. *J. Nutr.* **1999**, *129*, 1979–1983.
13. Tovar, A.R.; Santos, A.; Halhali, A.; Bourges, H.; Torres, N. Hepatic histidase gene expression responds to protein rehabilitation in undernourished growing rats. *J. Nutr.* **1998**, *128*, 1631–1635.
14. Kang-Lee, Y.A.; Harper, A.E. Effect of induction of histidase on histidine metabolism in vivo. *J. Nutr.* **1979**, *109*, 291–299.
15. Feigelson, M. Estrogenic regulation of hepatic histidase during postnatal development and adulthood. *J. Biol. Chem.* **1968**, *243*, 5088–5093.
16. Borsook, H.; Deasy, C.L.; Haagen-Smit, A.J.; Keighley, G.; Lowy, P.H. Metabolism of c14-labeled glycine, L-histidine, L-leucine, and L-lysine. *J. Biol. Chem.* **1950**, *187*, 839–848.
17. Wolf, G. The metabolism of alpha-C14-histidine in the intact rat. I. Radioactivity in amino acids from protein. *J. Biol. Chem.* **1953**, *200*, 637–645.
18. Harris, R.C.; Marlin, D.J.; Dunnett, M.; Snow, D.H.; Hultman, E. Muscle buffering capacity and dipeptide content in the thoroughbred horse, greyhound dog and man. *Comp. Biochem. Physiol. A* **1990**, *97*, 249–251, doi:10.1016/0300-9629(90)90180-z.
19. Guiotto, A.; Calderan, A.; Borin, P.R. and G. Carnosine and Carnosine-Related Antioxidants: A Review. Available online: http://www.eurekaselect.com/60152/article (accessed on 28 February 2018).
20. Asahi, R.; Tanaka, K.; Fujimi, T.J.; Kanzawa, N.; Nakajima, S. Proline decreases the suppressive effect of histidine on food intake and fat accumulation. *J. Nutr. Sci. Vitaminol. (Tokyo)* **2016**, *62*, 277–280, doi:10.3177/jnsv.62.277.
21. Xie, H.; He, S.-H. Roles of histamine and its receptors in allergic and inflammatory bowel diseases. *World J. Gastroenterol. WJG* **2005**, *11*, 2851–2857, doi:10.3748/wjg.v11.i19.2851.
22. Branco, A.C.C.C.; Yoshikawa, F.S.Y.; Pietrobon, A.J.; Sato, M.N. Role of histamine in modulating the immune response and inflammation. *Mediat. Inflamm.* **2018**, *2018*, 9524075, doi:10.1155/2018/9524075.
23. Sheiner, J.B.; Morris, P.; Anderson, G.H. Food intake suppression by histidine. *Pharmacol. Biochem. Behav.* **1985**, *23*, 721–726.

24. Vaziri, P.; Dang, K.; Anderson, G.H. Evidence for histamine involvement in the effect of histidine loads on food and water intake in rats. *J. Nutr.* **1997**, *127*, 1519–1526.
25. Zeitzer, J.M. Chapter Six—Control of Sleep and Wakefulness in Health and Disease. In *Progress in Molecular Biology and Translational Science*; Gillette, M.U., Ed.; Chronobiology: Biological Timing in Health and Disease; Academic Press: Cambridge, MA, USA, 2013; Volume 119, pp. 137–154.
26. Lin, J.S. Brain structures and mechanisms involved in the control of cortical activation and wakefulness, with emphasis on the posterior hypothalamus and histaminergic neurons. *Sleep Med. Rev.* **2000**, *4*, 471–503, doi:10.1053/smrv.2000.0116.
27. Simons, F.E.R. Advances in H1-antihistamines. *N. Engl. J. Med.* **2004**, *351*, 2203–2217, doi:10.1056/NEJMra033121.
28. Scammell, T.E.; Jackson, A.C.; Franks, N.P.; Wisden, W.; Dauvilliers, Y. Histamine: Neural circuits and new medications. *Sleep* **2019**, *42*, zsy183, doi:10.1093/sleep/zsy183.
29. Abe Details and Download Full Text PDF: Role of Histidine-Related Compounds as Intracellular Proton Buffering Constituents in Vertebrate Muscle. Available online: https://www.pubfacts.com/detail/10951092/Role-of-histidine-related-compounds-as-intracellular-proton-buffering-constituents-in-vertebrate-mus (accessed on 23 March 2018).
30. Abe, H. Role of histidine-related compounds as intracellular proton buffering constituents in vertebrate muscle. *Biochem. Biokhimiia* **2000**, *65*, 757–765.
31. Nair, N.G.; Perry, G.; Smith, M.A.; Reddy, V.P. NMR studies of zinc, copper, and iron binding to histidine, the principal metal ion complexing site of amyloid-β peptide. *J. Alzheimers Dis.* **2010**, *20*, 57–66, doi:10.3233/JAD-2010-1346.
32. Morris, P.J.; Bruce Martin, R. Stereoselective formation of cobalt(II), nickel(II) and zinc(II) chelates of histidine. *J. Inorg. Nucl. Chem.* **1970**, *32*, 2891–2897, doi:10.1016/0022-1902(70)80352-9.
33. Schmidt, J.A.; Rinaldi, S.; Scalbert, A.; Ferrari, P.; Achaintre, D.; Gunter, M.J.; Appleby, P.N.; Key, T.J.; Travis, R.C. Plasma concentrations and intakes of amino acids in male meat-eaters, fish-eaters, vegetarians and vegans: A cross-sectional analysis in the EPIC-Oxford cohort. *Eur. J. Clin. Nutr.* **2016**, *70*, 306–312, doi:10.1038/ejcn.2015.144.
34. Iwasaki, M.; Ishihara, J.; Takachi, R.; Todoriki, H.; Yamamoto, H.; Miyano, H.; Yamaji, T.; Tsugane, S. Validity of a self-administered food-frequency questionnaire for assessing amino acid intake in japan: comparison with intake from 4-day weighed dietary records and plasma levels. *J. Epidemiol.* **2016**, *26*, 36–44, doi:10.2188/jea.JE20150044.
35. Steele, B.F.; Le Bovit, C.B. Leucine and histidine tolerance in the human. *J. Nutr.* **1951**, *45*, 235–244.
36. Block, W.D.; Westhoff, M.H.; Steele, B.F. Histidine metabolism in the human adult: Histidine blood tolerance, and the effect of continued free L-histidine ingestion on the concentration of imidazole compounds in blood and urine. *J. Nutr.* **1967**, *91*, 189–194.
37. Henkin, R.I. New aspects in the control of food intake and appetite. *Ann. N. Y. Acad. Sci.* **1977**, *300*, 321–334, doi:10.1111/j.1749-6632.1977.tb19332.x.
38. Okubo, H.; Sasaki, S. Histidine intake may negatively correlate with energy intake in human: A cross-sectional study in Japanese female students aged 18 years. *J. Nutr. Sci. Vitaminol. (Tokyo)* **2005**, *51*, 329–334.
39. Nakajima, S.; Hamada, M.; Tsuchiya, T.; Okuda, H. Suppression of food intake by histidine-rich protein under low energy intake. *Nippon Eiyo Shokuryo Gakkaishi J. Jpn. Soc. Nutr. Food Sci.* **2000**, *53*, 207–214.
40. Schechter, P.J.; Prakash, N.J. Failure of oral L-histidine to influence appetite or affect zinc metabolism in man: A double-blind study. *Am. J. Clin. Nutr.* **1979**, *32*, 1011–1014.
41. Geliebter, A.A.; Hashim, S.A.; Van Itallie, T.B. Oral L-histidine fails to reduce taste and smell acuity but induces anorexia and urinary zinc excretion. *Am. J. Clin. Nutr.* **1981**, *34*, 119–120.
42. Sasahara, I.; Fujimura, N.; Nozawa, Y.; Furuhata, Y.; Sato, H. The effect of histidine on mental fatigue and cognitive performance in subjects with high fatigue and sleep disruption scores. *Physiol. Behav.* **2015**, *147*, 238–244, doi:10.1016/j.physbeh.2015.04.042.
43. Layrisse, M.; Martínez-Torres, C.; Leets, I.; Taylor, P.; Ramírez, J. Effect of histidine, cysteine, glutathione or beef on iron absorption in humans. *J. Nutr.* **1984**, *114*, 217–223.
44. Schölmerich, J.; Freudemann, A.; Köttgen, E.; Wietholtz, H.; Steiert, B.; Löhle, E.; Häussinger, D.; Gerok, W. Bioavailability of zinc from zinc-histidine complexes. I. Comparison with zinc sulfate in healthy men. *Am. J. Clin. Nutr.* **1987**, *45*, 1480–1486, doi:10.1093/ajcn/45.6.1480.

45. Yoganathan, S.; Sudhakar, S.V.; Arunachal, G.; Thomas, M.; Subramanian, A.; George, R.; Danda, S. Menkes disease and response to copper histidine: An Indian case series. *Ann. Indian Acad. Neurol.* **2017**, *20*, 62, doi:10.4103/0972-2327.199907.
46. Jain, P.; Kannan, L.; Chakrabarty, B.; Kumar, A.; Gupta, N.; Kabra, M.; Gulati, S. Menkes disease – An important cause of early onset refractory seizures. *J. Pediatr. Neurosci.* **2014**, *9*, 11–16, doi:10.4103/1817-1745.131471.
47. Christodoulou, J.; Danks, D.M.; Sarkar, B.; Baerlocher, K.E.; Casey, R.; Horn, N.; Tümer, Z.; Clarke, J.T. Early treatment of Menkes disease with parenteral copper-histidine: Long-term follow-up of four treated patients. *Am. J. Med. Genet.* **1998**, *76*, 154–164.
48. Yamane, T.; Inoue, Y.; Kobayashi-Hattori, K.; Oishi, Y. Effects of an Amino acid Deficiency on Hyaluronan Synthesis in Human Dermal Fibroblasts. *Food Sci. Technol. Res.* **2016**, *22*, 279–281, doi:10.3136/fstr.22.279.
49. Voorhees, J.J.; Chakrabarti, S.G.; Bernstein, I.A. The metabolism of "histidine-rich" protein in normal and psoriatic keratinization. *J. Invest Dermatol* **1968**, *51*, 344–354.
50. Tan, S.P.; Brown, S.B.; Griffiths, C.E.; Weller, R.B.; Gibbs, N.K. Feeding filaggrin: Effects of l-histidine supplementation in atopic dermatitis. *Clin. Cosmet. Investig. Dermatol.* **2017**, *10*, 403–411, doi:10.2147/CCID.S146760.
51. Li, Y.-C.; Li, C.-L.; Qi, J.-Y.; Huang, L.-N.; Shi, D.; Du, S.-S.; Liu, L.-Y.; Feng, R.-N.; Sun, C.-H. Relationships of dietary histidine and obesity in northern Chinese adults, an internet-based cross-sectional study. *Nutrients* **2016**, *8*, doi:10.3390/nu8070420.
52. Feng, R.N.; Niu, Y.C.; Sun, X.W.; Li, Q.; Zhao, C.; Wang, C.; Guo, F.C.; Sun, C.H.; Li, Y. Histidine supplementation improves insulin resistance through suppressed inflammation in obese women with the metabolic syndrome: A randomised controlled trial. *Diabetologia* **2013**, *56*, 985–994, doi:10.1007/s00125-013-2839-7.
53. Greenberg, A.S.; Obin, M.S. Obesity and the role of adipose tissue in inflammation and metabolism. *Am. J. Clin. Nutr.* **2006**, *83*, 461S–465S.
54. Lee, Y.; Hsu, C.; Lin, M.; Liu, K.; Yin, M. Histidine and carnosine delay diabetic deterioration in mice and protect human low density lipoprotein against oxidation and glycation. *Eur. J. Pharmacol.* **2005**, *513*, 145–150, doi:10.1016/j.ejphar.2005.02.010.
55. Ookuma, K.; Yoshimatsu, H.; Sakata, T.; Fujimoto, K.; Fukagawa, F. Hypothalamic sites of neuronal histamine action on food intake by rats. *Brain Res.* **1989**, *490*, 268–275.
56. Kasaoka, S.; Tsuboyama-Kasaoka, N.; Kawahara, Y.; Inoue, S.; Tsuji, M.; Ezaki, O.; Kato, H.; Tsuchiya, T.; Okuda, H.; Nakajima, S. Histidine supplementation suppresses food intake and fat accumulation in rats. *Nutrition* **2004**, *20*, 991–996, doi:10.1016/j.nut.2004.08.006.
57. Schwartz, J.C.; Lampart, C.; Rose, C. Histamine formation in rat brain in vivo: Effects of histidine loads. *J. Neurochem.* **1972**, *19*, 801–810.
58. Yoshimatsu, H.; Chiba, S.; Tajima, D.; Akehi, Y.; Sakata, T. Histidine suppresses food intake through its conversion into neuronal histamine. *Exp. Biol. Med.* **2002**, *227*, 63–68.
59. Holeček, M.; Vodeničarovová, V. Effects of histidine supplementation on amino acid metabolism in rats. *Physiol. Res.* **2020**, *69*, 99–111, doi:10.33549/physiolres.934296.
60. Rossi, D.J.; Brady, J.D.; Mohr, C. Astrocyte metabolism and signaling during brain ischemia. *Nat. Neurosci.* **2007**, *10*, 1377–1386, doi:10.1038/nn2004.
61. Yiu, G.; He, Z. Glial inhibition of CNS axon regeneration. *Nat. Rev. Neurosci.* **2006**, *7*, 617–627, doi:10.1038/nrn1956.
62. Liao, R.; Jiang, L.; Wang, R.; Zhao, H.; Chen, Y.; Li, Y.; Wang, L.; Jie, L.-Y.; Zhou, Y.; Zhang, X.; et al. Histidine provides long-term neuroprotection after cerebral ischemia through promoting astrocyte migration. *Sci. Rep.* **2015**, *5*, 15356, doi:10.1038/srep15356.
63. Zhao, Y.-Y.; Yuan, Y.; Chen, Y.; Jiang, L.; Liao, R.-J.; Wang, L.; Zhang, X.-N.; Ohtsu, H.; Hu, W.-W.; Chen, Z. Histamine promotes locomotion recovery after spinal cord hemisection via inhibiting astrocytic scar formation. *CNS Neurosci. Ther.* **2015**, *21*, 454–462, doi:10.1111/cns.12379.
64. Chen, Z.; Li, W.-D.; Zhu, L.-J.; Shen, Y.-J.; Wei, E.-Q. Effects of histidine, a precursor of histamine, on pentylenetetrazole-induced seizures in rats. *Acta Pharmacol. Sin.* **2002**, *23*, 361–366.
65. Wada Yuji et al. Biphasic action of the histamine precursor l-histidine in the rat kindling model of epilepsy. *Neurosci. Lett.* **1996**, *204*, 205–208, doi:10.1016/0304-3940(96)12358-2.

66. Kaneko, J.; Enya, A.; Enomoto, K.; Ding, Q.; Hisatsune, T. Anserine (beta-alanyl-3-methyl-L-histidine) improves neurovascular-unit dysfunction and spatial memory in aged AβPPswe/PSEN1dE9 Alzheimer's-model mice. *Sci. Rep.* **2017**, *7*, 12571, doi:10.1038/s41598-017-12785-7.
67. Solomon, J.K.; Geison, R.L. L-histidine-induced hypercholesterolemia: Characteristics of cholesterol biosynthesis in rat livers. *Proc. Soc. Exp. Biol. Med. Soc. Exp. Biol. Med.* **1978**, *159*, 44–47, doi:10.3181/00379727-159-40280.
68. Ohmura, E.; Aoyama, Y.; Yoshida, A. Changes in lipids in liver and serum of rats fed a histidine-excess diet or cholesterol-supplemented diets. *Lipids* **1986**, *21*, 748–753, doi:10.1007/bf02535406.
69. Harvey, P.W.; Hunsaker, H.A.; Allen, K.G. Dietary L-histidine-induced hypercholesterolemia and hypocupremia in the rat. *J. Nutr.* **1981**, *111*, 639–647, doi:10.1093/jn/111.4.639.
70. Muramatsu, K.; Odagiri, H.; Morishita, S.; Takeuchi, H. Effect of excess levels of individual amino acids on growth of rats fed casein diets. *J. Nutr.* **1971**, *101*, 1117–1125, doi:10.1093/jn/101.9.1117.
71. Benevenga, N.J.; Steele, R.D. Adverse effects of excessive consumption of amino acids. *Annu. Rev. Nutr.* **1984**, *4*, 157–181, doi:10.1146/annurev.nu.04.070184.001105.
72. Schirmer, M.D.; Harper, A.E. Adaptive responses of mammalian histidine-degrading enzymes. *J. Biol. Chem.* **1970**, *245*, 1204–1211.
73. Harper, A.E.; Becker, R.V.; Stucki, W.P. Some effects of excessive intakes of indispensable amino acids. *Proc. Soc. Exp. Biol. Med. Soc. Exp. Biol. Med.* **1966**, *121*, 695–699, doi:10.3181/00379727-121-30863.
74. Ikezaki, S.; Nishikawa, A.; Furukawa, F.; Imazawa, T.; Enami, T.; Mitsui, M.; Takahashi, M. 13-week subchronic toxicity study of L-histidine monohydrochloride in F344 rats. *Eisei Shikenjo Hokoku* **1994**, 57–63.
75. Sun, X.; Feng, R.; Li, Y.; Lin, S.; Zhang, W.; Li, Y.; Sun, C.; Li, S. Histidine supplementation alleviates inflammation in the adipose tissue of high-fat diet-induced obese rats via the NF-κB- and PPARγ-involved pathways. *Br. J. Nutr.* **2014**, *112*, 477–485, doi:10.1017/S0007114514001056.
76. Snedeker, S.M.; Greger, J.L. Metabolism of Zinc, Copper and Iron as Affected by Dietary Protein, Cysteine and Histidine. *J. Nutr.* **1983**, *113*, 644–652, doi:10.1093/jn/113.3.644.
77. Van Campen, D.; Gross, E. Effect of Histidine and Certain Other Amino Acids on the Absorption of Iron-59 by Rats. *J. Nutr.* **1969**, *99*, 68–74, doi:10.1093/jn/99.1.68.
78. Darwish, H.M.; Cheney, J.C.; Schmitt, R.C.; Ettinger, M.J. Mobilization of copper(II) from plasma components and mechanisms of hepatic copper transport. *Am. J. Physiol.* **1984**, *246*, G72–G79, doi:10.1152/ajpgi.1984.246.1.G72.
79. Hartter, D.E.; Barnea, A. Brain tissue accumulates 67copper by two ligand-dependent saturable processes. A high affinity, low capacity and a low affinity, high capacity process. *J. Biol. Chem.* **1988**, *263*, 799–805.
80. Mas, A.; Sarkar, B. Uptake of 67Cu by isolated human trophoblast cells. *Biochim. Biophys. Acta BBA-Mol. Cell Res.* **1992**, *1135*, 123–128, doi:10.1016/0167-4889(92)90127-W.
81. McArdle, H.J.; Guthrie, J.R.; Ackland, M.L.; Danks, D.M. Albumin has no role in the uptake of copper by human fibroblasts. *J. Inorg. Biochem.* **1987**, *31*, 123–131, doi:10.1016/0162-0134(87)80057-0.
82. Freeman, R.M.; Taylor, P.R. Influence of histidine administration on zinc metabolism in the rat. *Am. J. Clin. Nutr.* **1977**, *30*, 523–527.
83. Buxani-Rice, S.; Ueda, F.; Bradbury, M. W. B. Transport of Zinc-65 at the Blood-Brain Barrier During Short Cerebrovascular Perfusion in the Rat: Its Enhancement by Histidine. *J. Neurochem.* **2002**, *62*, 665–672, doi:10.1046/j.1471-4159.1994.62020665.x.
84. Buxani-Rice, S.; Ueda, F.; Bradbury, M.W. Transport of zinc-65 at the blood-brain barrier during short cerebrovascular perfusion in the rat: Its enhancement by histidine. *J. Neurochem.* **1994**, *62*, 665–672.
85. Wensink, J.; Van den Hamer, C.J. Effect of excess dietary histidine on rate of turnover of 65Zn in brain of rat. *Biol. Trace Elem. Res.* **1988**, *16*, 137–150, doi:10.1007/BF02797098.
86. Van Wouwe, J.P.; Hoogenkamp, S.; Van den Hamer, C.J. A histidine supplement and regulation of the zinc status in Swiss random mice. *Biol. Trace Elem. Res.* **1990**, *24*, 207–216.
87. Frezza, C. Histidine metabolism boosts cancer therapy. *Nature* **2018**, *559*, 484–485, doi:10.1038/d41586-018-05573-4.
88. Kanarek, N.; Keys, H.R.; Cantor, J.R.; Lewis, C.A.; Chan, S.H.; Kunchok, T.; Abu-Remaileh, M.; Freinkman, E.; Schweitzer, L.D.; Sabatini, D.M. Histidine catabolism is a major determinant of methotrexate sensitivity. *Nature* **2018**, *559*, 632–636, doi:10.1038/s41586-018-0316-7.

89. Ikezaki, S.; Nishikawa, A.; Furukawa, F.; Enami, T.; Mitsui, M.; Tanakamaru, Z.; Kim, H.C.; Lee, I.S.; Imazawa, T.; Takahashi, M. Long-term toxicity/carcinogenicity study of L-histidine monohydrochloride in F344 rats. *Food Chem. Toxicol. Int. J. Publ. Br. Ind. Biol. Res. Assoc.* **1996**, *34*, 687–691.
90. Gullino, P.; Winitz, M.; Birnbaum, S.M.; Cornfield, J.; Otey, M.C.; Greenstein, J.P. Studies on the metabolism of amino acids and related compounds in vivo. I. Toxicity of essential amino acids, individually and in mixtures, and the protective effect of l-arginine. *Arch. Biochem. Biophys.* **1956**, *64*, 319–332, doi:10.1016/0003-9861(56)90276-4.
91. Figueroa, J.L.; Lewis, A.J.; Miller, P.S.; Fischer, R.L.; Diedrichsen, R.M. Growth, carcass traits, and plasma amino acid concentrations of gilts fed low-protein diets supplemented with amino acids including histidine, isoleucine, and valine,. *J. Anim. Sci.* **2003**, *81*, 1529–1537, doi:10.2527/2003.8161529x.
92. Park, S.W.; Kim, C.H.; Kim, J.W.; Shin, H.S.; Paik, I.K.; Kil, D.Y. Effect of dietary supplementation of blood meal and additional magnesium on carnosine and anserine concentrations of pig muscles. *Korean J. Food Sci. Anim. Resour.* **2014**, *34*, 252–256, doi:10.5851/kosfa.2014.34.2.252.
93. Edmonds, M.S.; Baker, D.H. Comparative effects of individual amino acid excesses when added to a corn-soybean meal diet: Effects on growth and dietary choice in the chick. *J. Anim. Sci.* **1987**, *65*, 699–705.
94. Auh, J.; Namgung, N.; Shin, K.; Park, S.; Paik, I. Effects of supplementary blood meal on the content of carnosine and anserine in broiler meat. *J. Poult. Sci.* **2010**, *47*, 302–309, doi:10.2141/jpsa.010024.
95. Park, S.W.; Kim, C.H.; Namgung, N.; Jung, B.Y.; Paik, I.K.; Kil, D.Y. Effects of dietary supplementation of histidine, β-alanine, magnesium oxide, and blood meal on carnosine and anserine concentrations of broiler breast meat. *J. Poult. Sci.* **2013**, *50*, 251–256, doi:10.2141/jpsa.0120064.
96. Kralik, G.; Sak-Bosnar, M.; Kralik, Z.; Galović, O.; Grčević, M.; Kralik, I. Effect of β-alanine and l-histidine on concentration of carnosine in muscle tissue and oxidative stability of chicken meat. *Poljoprivreda* **2015**, *21*, 190–194, doi:10.18047/poljo.21.1.sup.45.
97. Haug, A.; R∅dbotten, R.; Mydland, L.T.; Christophersen, O.A. Increased broiler muscle carnosine and anserine following histidine supplementation of commercial broiler feed concentrate. *Acta Agric. Scand. Sect.-Anim. Sci.* **2008**, *58*, 71–77, doi:10.1080/09064700802213545.
98. Kai, S.; Watanabe, G.; Kubota, M.; Kadowaki, M.; Fujimura, S. Effect of dietary histidine on contents of carnosine and anserine in muscles of broilers. *Anim. Sci. J. Nihon Chikusan Gakkaiho* **2015**, *86*, 541–546, doi:10.1111/asj.12322.
99. Kopeć, W.; Jamroz, D.; Wiliczkiewicz, A.; Biazik, E.; Pudlo, A.; Hikawczuk, T.; Skiba, T.; Korzeniowska, M. Influence of different histidine sources and zinc supplementation of broiler diets on dipeptide content and antioxidant status of blood and meat. *Br. Poult. Sci.* **2013**, *54*, 454–465, doi:10.1080/00071668.2013.793295.
100. Jiang, W.-D.; Qu, B.; Feng, L.; Jiang, J.; Kuang, S.-Y.; Wu, P.; Tang, L.; Tang, W.-N.; Zhang, Y.-A.; Zhou, X.-Q.; et al. Histidine prevents cu-induced oxidative stress and the associated decreases in mrna from encoding tight junction proteins in the intestine of grass carp (Ctenopharyngodon idella). *PLoS ONE* **2016**, *11*, e0157001, doi:10.1371/journal.pone.0157001.
101. Wilson, R.P.; Poe, W.E.; Robinson, E.H. Leucine, isoleucine, valine and histidine requirements of fingerling channel catfish. *J. Nutr.* **1980**, *110*, 627–633.
102. Michelato, M.; Zaminhan, M.; Boscolo, W.R.; Nogaroto, V.; Vicari, M.; Artoni, R.F.; Furuya, V.R.B.; Furuya, W.M. Dietary histidine requirement of Nile tilapia juveniles based on growth performance, expression of muscle-growth-related genes and haematological responses. *Aquaculture* **2017**, *467*, 63–70, doi:10.1016/j.aquaculture.2016.06.038.
103. Abe, H.; Dobson, G.P.; Hoeger, U.; Parkhouse, W.S. Role of histidine-related compounds to intracellular buffering in fish skeletal muscle. *Am. J. Physiol.* **1985**, *249*, R449–R454, doi:10.1152/ajpregu.1985.249.4.R449.
104. Breck, O.; Bjerk\a as, E.; Campbell, P.; Arnesen, P.; Haldorsen, P.; Waagbø, R. Cataract preventative role of mammalian blood meal, histidine, iron and zinc in diets for Atlantic salmon (Salmo salar L.) of different strains. *Aquac. Nutr.* **2003**, *9*, 341–350.
105. Bjerkås, E.; Breck, O.; Waagbo, R. The role of nutrition in cataract formation in farmed fish. *CAB Rev. Perspect. Agric. Vet. Sci. Nutr. Nat. Resour.* **2006**, *1*, doi:10.1079/PAVSNNR20061033
106. Remø, S.C.; Olsvik, P.A.; Torstensen, B.E.; Amlund, H.; Breck, O.; Waagbø, R. Susceptibility of Atlantic salmon lenses to hydrogen peroxide oxidation ex vivo after being fed diets with vegetable oil and methylmercury. *Exp. Eye Res.* **2011**, *92*, 414–424, doi:10.1016/j.exer.2011.02.018.
107. Remø, S.C.; Hevrøy, E.M.; Olsvik, P.A.; Fontanillas, R.; Breck, O.; Waagbø, R. Dietary histidine requirement to reduce the risk and severity of cataracts is higher than the requirement for growth in Atlantic salmon

smolts, independently of the dietary lipid source. *Br. J. Nutr.* **2014**, *111*, 1759–1772, doi:10.1017/S0007114513004418.
108. Trösse, C.; Waagbø, R.; Breck, O.; Stavrum, A.-K.; Petersen, K.; Olsvik, P.A. Genome-wide transcription analysis of histidine-related cataract in Atlantic salmon (Salmo salar L). *Mol. Vis.* **2009**, *15*, 1332–1350.
109. Waagbø, R.; Trösse, C.; Koppe, W.; Fontanillas, R.; Breck, O. Dietary histidine supplementation prevents cataract development in adult Atlantic salmon, *Salmo salar* L., in seawater. *Br. J. Nutr.* **2010**, *104*, 1460–1470, doi:10.1017/S0007114510002485.
110. Schoof, A.; Gabel, M.; Voigt, J.; Schönhusen, U.; Kluth, H. Investigations on the influence of duodenal histidine infusion on nitrogen and amino acid turnover of growing German Holstein bulls. *Arch. Tierernahr.* **2000**, *53*, 303–321.
111. Union, P.O. of the E. Feed Evaluation and Protein Requirement Systems for Ruminants. Available online: https://publications.europa.eu/en/publication-detail/-/publication/72fd322e-2083-4a19-9105-758eda4faf9b/language-en (accessed on 2 March 2018).
112. Gabel, M.; Poppe, S. Intestinal amino acid in supply in relation to the amino acid requirements in growing bulls. *Arch. Tierernahr.* **1986**, *36*, 227–234.
113. Gao, H.; Hu, H.; Zheng, N.; Wang, J. Leucine and histidine independently regulate milk protein synthesis in bovine mammary epithelial cells via mTOR signaling pathway. *J. Zhejiang Univ. Sci. B* **2015**, *16*, 560–572, doi:10.1631/jzus.B1400337.
114. Prizant, R.L.; Barash, I. Negative effects of the amino acids Lys, His, and Thr on S6K1 phosphorylation in mammary epithelial cells. *J. Cell. Biochem.* **2008**, *105*, 1038–1047, doi:10.1002/jcb.21904.
115. Toerien, C.A.; Trout, D.R.; Cant, J.P. Nutritional stimulation of milk protein yield of cows is associated with changes in phosphorylation of mammary eukaryotic initiation factor 2 and ribosomal s6 kinase 1. *J. Nutr.* **2010**, *140*, 285–292, doi:10.3945/jn.109.114033.
116. Doelman, J.; Purdie, N.G.; Osborne, V.R.; Cant, J.P. Short communication: The effects of histidine-supplemented drinking water on the performance of lactating dairy cows. *J. Dairy Sci.* **2008**, *91*, 3998–4001, doi:10.3168/jds.2008-1131.
117. Hadrová, S.; Krizova, L.; Richter, M.; Třináctý, J.; Dračková, M. The effect of duodenal infusion of histidine on milk yield, milk composition, and plasma amino acids in dairy cows. *J. Anim. Feed Sci.* **2012**, *21*, 555–565, doi:10.22358/jafs/66129/2012.

© 2020 by the authors. Submitted for possible open access publication under the terms and conditions of the Creative Commons Attribution (CC BY) license (http://creativecommons.org/licenses/by/4.0/).
